# Supplementary material for: Optimal Transport Distances to Characterize Electronic Excitations
Source: J Chem Theory Comput. 2024 Jun 14;20(13):5635–42. doi: 10.1021/acs.jctc.4c00289 (PMC11238536; doi:10.1021/acs.jctc.4c00289)
Supplement: Supplementary file 1 — ct4c00289_si_001.pdf [file ct4c00289_si_001.pdf]

Supporting information for "Optimal transport distances to characterise electronic excitations"

Annina Z. Lieberherr,[*,†] Paola Gori-Giorgi,[‡,¶] and Klaas J. H. Giesbertz[‡]

†*Department of Chemistry, University of Oxford, Physical and Theoretical Chemistry Laboratory, South Parks Road, Oxford OX1 3QZ, United Kingdom*

‡*Department of Chemistry and Pharmaceutical Sciences, Amsterdam Institute of Molecular and Life Sciences (AIMMS), Faculty of Science, Vrije Universiteit Amsterdam, De Boelelaan 1083, 1081HV Amsterdam, The Netherlands*

¶*Microsoft Research AI for Science, Evert van de Beekstraat 354, 1118CZ Schiphol, The Netherlands*

E-mail: annina.lieberherr@chem.ox.ac.uk

Contents

Molecules 2

Convergence with respect to entropic regularisation 3

Code 3

References **3**

Molecules

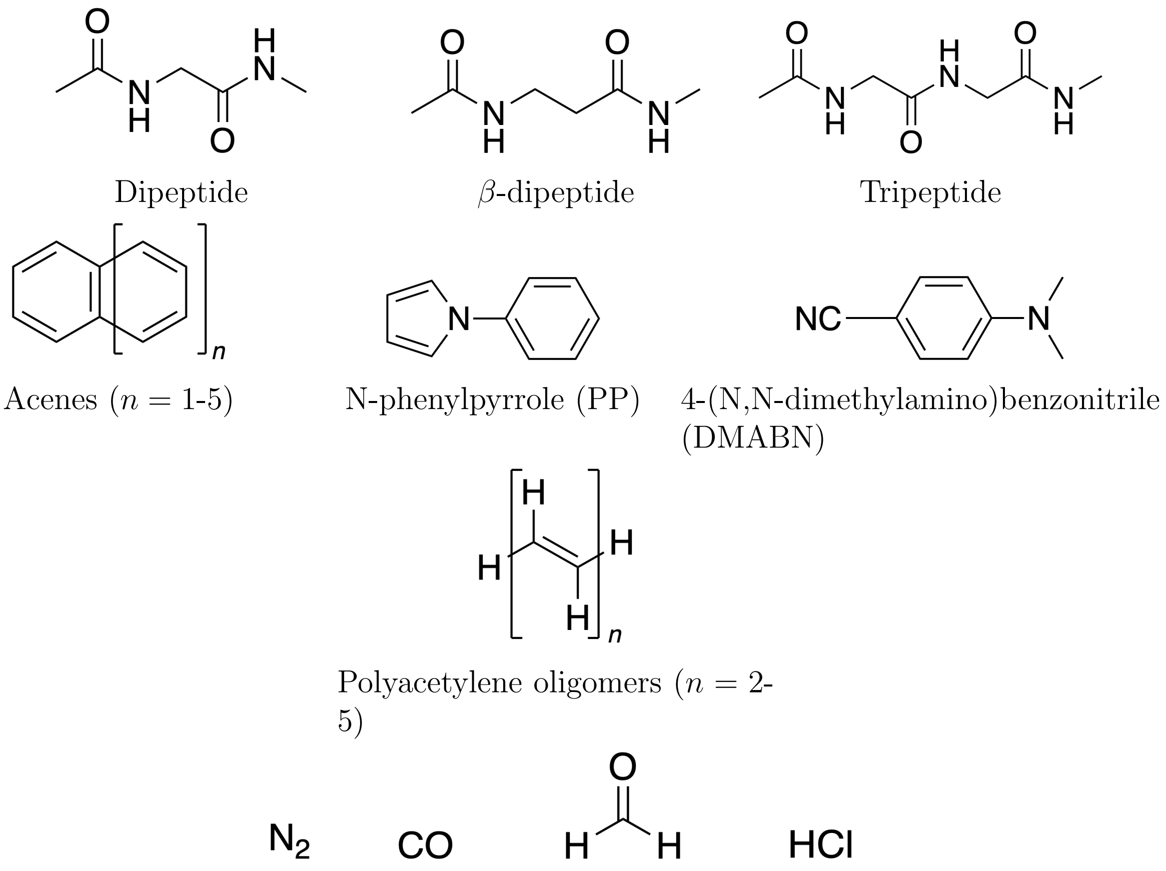

Figure S1: Molecules studied by Peach *et al.* and here.[1]

Convergence with respect to entropic regularisation

Table S1: Convergence of Θ with regularisation parameter $\varepsilon = 10^{-\sigma} d_{\max}$, where $d_{\max}$ is the maximum possible distance for a molecular grid. The dipeptide shows the trend for the cc-pVTZ basis set, the N_2 molecule for the *d*-aug-cc-pVTZ basis set.

Excitation	Functional	σ			
		3	4	5	6
dipeptide, $n_1 \rightarrow \pi_2^*$	PBE	2**7.93**	28.4**7**	28.48	28.48
	B3LYP	16.**22**	16.7**7**	16.78	16.78
	CAM-B3LYP	20.**33**	20.9**0**	20.92	20.92
N_2, $^1\Pi_u$	PBE	**12.64**	17.**61**	17.82	17.82
	B3LYP	1**2.72**	**18.89**	19.1**5**	19.16
	CAM-B3LYP	**17.05**	24.**62**	24.90	24.90

Code

The code used to obtain the results in this study can be found at the github repository alieberherr/OTdensities.

References

(1) Peach, M. J. G.; Benfield, P.; Helgaker, T.; Tozer, D. J. Excitation energies in density functional theory: An evaluation and a diagnostic test. *J. Chem. Phys.* **2008**, *128*, 044118.
